# Association of the Cold Shock DEAD-Box RNA Helicase RhlE to the RNA Degradosome in Caulobacter crescentus

**DOI:** 10.1128/JB.00135-17

**Published:** 2017-06-13

**Authors:** Angel A. Aguirre, Alexandre M. Vicente, Steven W. Hardwick, Daniela M. Alvelos, Ricardo R. Mazzon, Ben F. Luisi, Marilis V. Marques

**Affiliations:** a Departamento de Microbiologia, Instituto de Ciências Biomédicas, Universidade de São Paulo, São Paulo, Brazil; b Department of Biochemistry, University of Cambridge, Cambridge, United Kingdom; Princeton University

**Keywords:** DEAD-box RNA helicase, RhlE, Caulobacter crescentus, RNA degradosome, cold shock

## Abstract

In diverse bacterial lineages, multienzyme assemblies have evolved that are central elements of RNA metabolism and RNA-mediated regulation. The aquatic Gram-negative bacterium Caulobacter crescentus, which has been a model system for studying the bacterial cell cycle, has an RNA degradosome assembly that is formed by the endoribonuclease RNase E and includes the DEAD-box RNA helicase RhlB. Immunoprecipitations of extracts from cells expressing an epitope-tagged RNase E reveal that RhlE, another member of the DEAD-box helicase family, associates with the degradosome at temperatures below those optimum for growth. Phenotype analyses of *rhlE*, *rhlB*, and *rhlE rhlB* mutant strains show that RhlE is important for cell fitness at low temperature and its role may not be substituted by RhlB. Transcriptional and translational fusions of *rhlE* to the *lacZ* reporter gene and immunoblot analysis of an epitope-tagged RhlE indicate that its expression is induced upon temperature decrease, mainly through posttranscriptional regulation. RNase E pulldown assays show that other proteins, including the transcription termination factor Rho, a second DEAD-box RNA helicase, and ribosomal protein S1, also associate with the degradosome at low temperature. The results suggest that the RNA degradosome assembly can be remodeled with environmental change to alter its repertoire of helicases and other accessory proteins.

**IMPORTANCE** DEAD-box RNA helicases are often present in the RNA degradosome complex, helping unwind secondary structures to facilitate degradation. Caulobacter crescentus is an interesting organism to investigate degradosome remodeling with change in temperature, because it thrives in freshwater bodies and withstands low temperature. In this study, we show that at low temperature, the cold-induced DEAD-box RNA helicase RhlE is recruited to the RNA degradosome, along with other helicases and the Rho protein. RhlE is essential for bacterial fitness at low temperature, and its function may not be complemented by RhlB, although RhlE is able to complement for *rhlB* loss. These results suggest that RhlE has a specific role in the degradosome at low temperature, potentially improving adaptation to this condition.

## INTRODUCTION

The complex structures that RNA can adopt underlie the capacity of the nucleic acid to regulate diverse cellular processes. RNA structure is likely to be in dynamic equilibrium in living cells, and when altered by environmental changes or in response to signals, the cell must quickly respond by ensuring that no misfolded RNA molecules accumulate. One such environmental condition is low temperature, which causes an increase in RNA secondary structure stability that could lead to altered function, particularly of mRNAs. A common response to cold temperature is the induction of RNA binding proteins and enzymes, such as the small cold shock proteins (CSPs), RNA helicases, and ribonucleases (RNases), which prevent or unwind secondary structures and turnover RNA and are important for cold adaptation (reviewed in reference 1; see also reference 2).

RNA helicases are important not only for stress response but also for basal RNA metabolism, being involved in the modulation of mRNA translation and degradation and in ribosome biogenesis (3, 4). The DEXD/H ATP-dependent RNA helicases belong to superfamily 2 of nucleic acid helicases (5, 6), and multiple paralogues are found in diverse genomes, particularly those of the Proteobacteria (7–9). The best functionally characterized bacterial DEAD-box RNA helicases are from Escherichia coli, which contains five paralogues: *csdA* (formerly *deaD*), *dbpA*, *rhlB*, *rhlE*, and *srmB*. These enzymes have specific functional roles: SrmB and CsdA participate in ribosome biogenesis (10–12), RhlB is involved in mRNA degradation (13, 14), DbpA has helicase activity dependent on 23S rRNA (15–17), and RhlE aids in the degradation of mRNA by polynucleotide phosphorylase (PNPase) and seems to regulate the roles of other helicases in ribosome maturation (18, 19).

In several bacteria, including E. coli, a large enzymatic complex responsible for RNA decay, the RNA degradosome, is assembled by protein-protein interactions using the carboxy-terminal domain of the enzyme RNase E as a scaffold (20). The compositions of the RNA degradosomes from several bacteria have been determined, and it has been shown that these assemblies typically contain an endoribonuclease (RNase E in most cases), a helicase (DEAD-box RNA helicase, in some cases Rho), an exoribonuclease (PNPase or RNase R), and a metabolic enzyme (enolase, aconitase, and phosphofructokinase) (21). Despite the variation in composition found among the degradosomes from members of different genera, a shared feature appears to be the presence of a DEAD-box RNA helicase, and in some organisms, multiple helicases have been shown to be able to interact with RNase E. The ubiquity of helicase proteins in degradosome assemblies highlights the importance of RNA unwinding or remodeling for degradation. It has been proposed that the ATP-dependent helicase activity of the DEAD-box helicases is necessary to improve RNA processing by RNase E and PNPase (13, 22–24). Under some conditions, such as cold shock, distinct DEAD-box RNA helicases were shown to associate with the degradosome in E. coli (25), suggesting that at low temperature, additional or more efficient enzymes are needed.

The genome of the free-living alphaproteobacterium Caulobacter crescentus NA1000 encodes four DEAD-box RNA helicases: CCNA_00878 (encoded by *rhlE*), CCNA_01546, CCNA_01923 (encoded by *rhlB*), and CCNA_02121 (26). CCNA_02121 has previously been shown to be regulated by the LexA repressor protein and probably takes part in the DNA damage SOS response (27). The *rhlB* gene encodes the main DEAD-box RNA helicase present in the C. crescentus RNA degradosome in cells grown at 30°C (28). In previous work, we showed that a transposon insertion into *rhlE* caused C. crescentus to become deficient in growth at low temperature and freezing survival (29), indicating that this helicase may have a role in stress response. Additionally, saturating transposon mutagenesis in the C. crescentus NA1000 genome indicated that *rhlE* is important for cellular fitness, even at 30°C (30).

In this work, we have characterized the *rhlE* gene, showing that it is highly induced at low temperature by both transcriptional and posttranscriptional mechanisms. We show that an *rhlE* knockout mutant has a severe cold-sensitive phenotype, and that it is not complemented by overexpressing the homologous *rhlB*. RhlE was identified to associate with the RNA degradosome at low temperature in an RhlB-independent manner. Other proteins that are recruited to the RNA degradosome at low temperature were also identified, showing that other enzymes may be required to facilitate the RNA degradation process under this condition.

## RESULTS

### Interplay between *rhlE* and *rhlB* for growth at low temperature.

**FIG 1:**
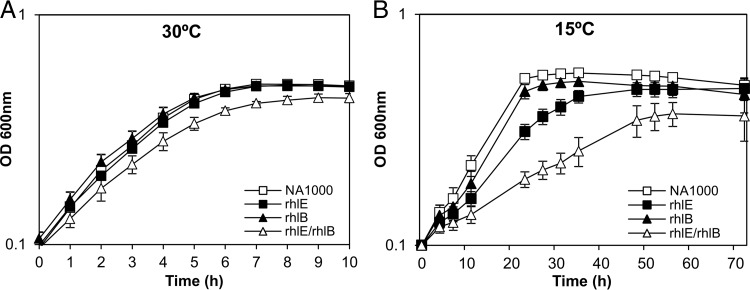
Growth of C. crescentus RNA helicase mutant strains at low temperature. The cultures were grown in PYE medium, and growth was monitored by measuring the OD at different time points. The following strains were used: NA1000, MM74 (rhlE), MM50 (rhlB), and MM82 (rhlE/rhlB). The curves are the means of the results from four experiments, and the standard deviation is indicated by vertical bars.

**FIG 2:**
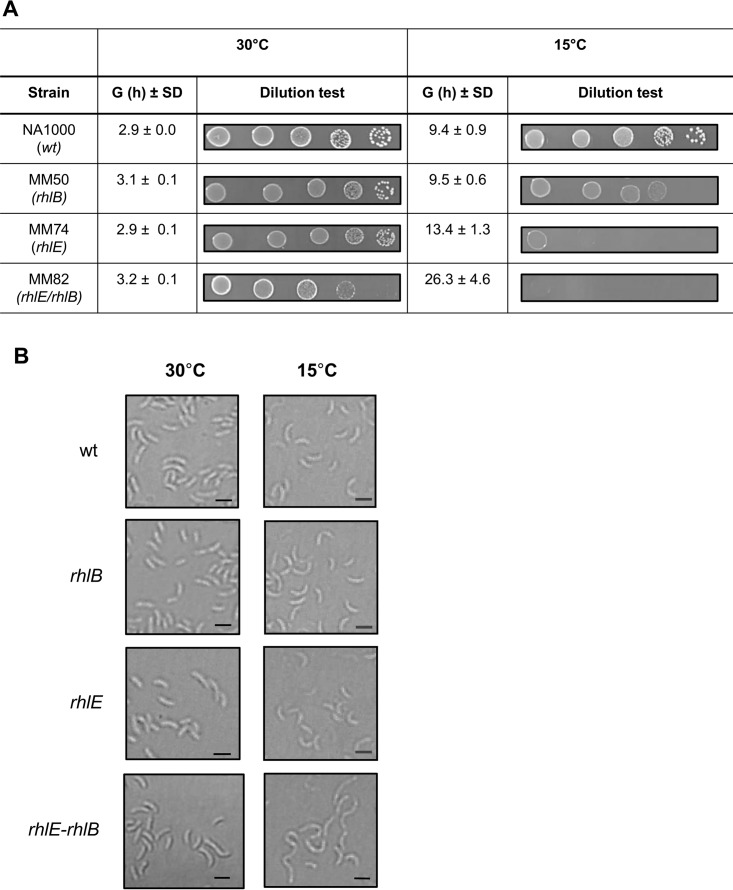
Cell viability and morphology of C. crescentus RNA helicase mutant strains at low temperature. (A) Serial dilutions of the cultures at an OD of 0.1 (10^−1^ to 10^−5^ in 10 μl) were plated in PYE medium and plates were incubated at the indicated temperatures. All strains were incubated at 15°C for 5 days, except for MM82, which was incubated at 15°C for 8 days. The average generation times (G) for four replicates of each culture with the respective standard deviation (SD) are indicated. (B) Cultures were grown in PYE medium at either 30°C or 15°C up to mid-log phase, and the cell morphology was evaluated by light microscopy. The strains used are as follows: NA1000 (wt), MM50 (*rhlB*), MM74 (*rhlE*), and MM82 (*rhlE-rhlB*). Bars indicate 2 μm.

The *rhlE* gene of C. crescentus was previously identified as necessary for freezing resistance following disruption of this gene with a mini-Tn*5* insertion (29), indicating that the RNA helicase RhlE is important for low-temperature adaptation, while the RhlB helicase was shown to be part of the RNA degradosome (28). In order to evaluate the roles of both enzymes in cell growth under this condition, a deletion mutant for the *rhlB* gene was constructed and combined with the *rhlE* knockout to generate an *rhlE rhlB* double mutant strain. The growth of each individual mutant and the double mutant at both 30°C and 15°C was monitored (Fig. 1). The *rhlB* and *rhlE* mutants displayed a growth pattern similar to that of the wild-type NA1000 strain at 30°C, while the double mutant displayed a slight reduction in growth, indicating that the loss of both RNA helicases is mildly deleterious to cells even at normal temperature (Fig. 1A). At 15°C, differences in the growth phenotypes were accentuated, especially for the strains carrying the *rhlE* mutation (Fig. 1B). These differences were determined more precisely by measuring the generation times of each strain (Fig. 2A), with the *rhlE* and *rhlE rhlB* mutants showing generation times that are 1.5 times and 2.7 times longer, respectively, than that of the wild-type (wt) strain at 15°C.

As the growth curves were determined by measuring the optical density, the results could be affected by the cell volume and not truly represent the viability of the mutant cells. In order to evaluate cell viability, growth was determined by CFU counting of serial dilutions (Fig. 2A). We can observe that the individual mutants grow well at 30°C, but the *rhlE* mutant shows smaller colonies (Fig. 2A, left). However, it still had a CFU count equivalent to that of the wild type, indicating that cells are viable to the same extent, but cell division is slower. The *rhlB* mutant presented colonies smaller than those of the wild type only at 15°C. On the other hand, the *rhlE rhlB* double mutant showed a reduction in CFU counts at 30°C and almost no growth at 15°C, even after 8 days (Fig. 2A, bottom). To assess the mutants' cell morphology, the cultures were examined by microscopy (Fig. 2B). The individual mutants showed no distinct phenotype at either 30°C or 15°C, but the double-mutant cells were more elongated at 15°C, indicating a severe cell division defect, consistent with the diminished viability observed. The growth complementation phenotypes described above were also confirmed by CFU counts (see Fig. S1 in the supplemental material).

**FIG 3:**
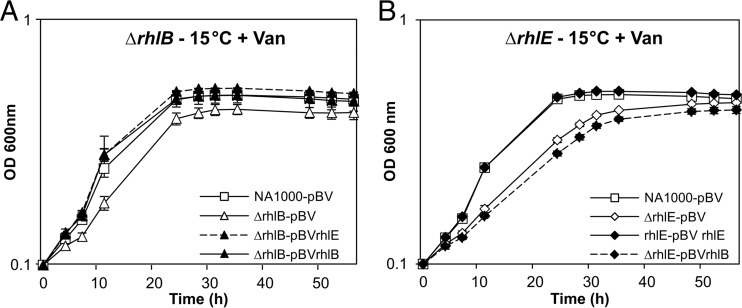
Complementation of C. crescentus mutant growth deficiency at low temperature. Δ*rhlB* (MM50) (A) and *rhlE*::mini-Tn*5* (MM74) (B) mutant strains harboring either the empty vector pBV-MCS4 (pBV) or the vector containing a copy of the indicated gene were grown in PYE-gentamicin medium containing 0.5 mM vanillate (Van) at 15°C, and growth was monitored by measuring the OD at different time points. The wild-type NA1000 strain harboring the empty vector pBV-MCS4 (NA1000-pBV) was included as a control. The curves represent the means of results from three experiments, and the standard deviation is indicated by vertical bars.

To explore if there is cross-complementation between these two helicases, the genes were provided in *trans* in the pBV vector under the control of a vanillate-inducible promoter. Growth in the presence of vanillate at 15°C of the strains carrying the pBV complementing vectors was compared with that of strains carrying the empty vector as controls. As shown in Fig. 3A, growth of the *rhlB* mutant at low temperature could be restored by a copy of the *rhlB* gene in *trans*, as well as a copy of the *rhlE* gene, indicating that there is a functional overlap of the two enzymes. However, in the converse experiments, the plasmid-carried *rhlB* gene did not complement the *rhlE* null strain at low temperature (Fig. 3B). Although it was not possible to quantify the expression from each construct, pBV-*rhlE* and pBV-*rhlB*, besides complementing their own gene loss, were equally able to complement the growth defect of the ΔCCNA_01546 strain, indicating that the genes were sufficiently expressed. These results suggest that while RhlE can substitute for RhlB function, the role of RhlE in cell growth at low temperature is not fulfilled by RhlB.

### RhlE is induced at low temperature.

**FIG 4:**
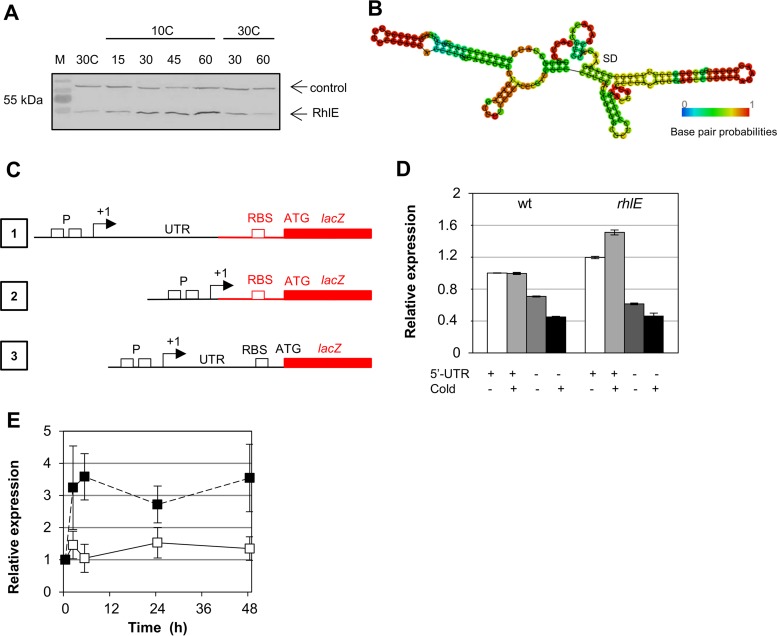
Regulation of RhlE in response to low temperature. (A) C. crescentus strain MM84, containing a tagged FLAG-RhlE protein, was incubated at 30°C to mid-log phase and then transferred to 10°C for 1 h. After this time, the culture was returned to 30°C for 2 h. Aliquots were taken at the indicated times, and protein accumulation was evaluated sequentially by immunoblotting using an anti-FLAG antibody and the anti-Fur antiserum. The arrows indicate the RhlE protein and a nonspecific protein that reacts with the anti-Fur serum as a control. M, prestained molecular mass marker. (B) Minimal free energy prediction of secondary structure of the *rhlE* 5′ UTR. (C) Schematic representation of the transcriptional and translational fusions to *lacZ*: 1, transcriptional fusion in pRK*lacZ*290 containing the promoter region (P) and the 5′ UTR; 2, transcriptional fusion in pRK*lacZ*290 containing only the promoter region; 3, translational fusion in pJBZ281 containing the promoter region, the 5′ UTR, the ribosome binding site (RBS), and the first codon of RhlE fused to *lacZ* in frame. In black are the elements that came from the *rhlE* locus, and in red are the vector sequences. (D) Expression of transcriptional fusions of the *rhlE* promoter in the presence or absence of the 5′ UTR to the *lacZ* reporter gene. Cultures were grown at 30°C to mid-log phase and then incubated at 10°C for 6 h, and expression was determined by β-galactosidase activity assays at these times. Results shown are the means of results from two experiments, and the range is indicated by vertical bars. (E) C. crescentus NA1000 harboring a translational fusion of the *rhlE* promoter and 5′ UTR to the *lacZ* reporter gene was grown at 30°C up to mid-log phase. Culture samples continued to grow at 30°C (white squares) or were incubated at 10°C (black squares), and expression was determined by β-galactosidase activity assays at the times indicated. Results shown are the means of results from two experiments, and standard deviation is indicated by vertical bars.

Since RhlE is necessary for optimal growth at low temperature, we constructed a strain carrying an epitope-tagged FLAG-RhlE in the original *rhlE* locus to determine RhlE expression in response to stress by immunoblotting. The expression was not induced by osmotic shock (using either NaCl or sucrose) or oxidative stress (potassium dichromate or paraquat) but increased when cells were grown at low temperature (Fig. S2). The FLAG-RhlE protein accumulation upon incubation at low temperature reached maximal levels after 2 h at 10°C, with no further increase observed when cold incubation was continued until 5 h (Fig. S3). To further explore the cold induction of *rhlE*, the cells were grown at 30°C and then transferred to 10°C for 60 min, returning to 30°C for another 90 min (Fig. 4A). Aliquots were taken at several times, and the protein levels were determined by immunoblotting with an anti-FLAG antibody against total cell extracts. Upon return to 30°C, the levels of FLAG-RhlE in the cell cultures rapidly returned to their original levels, suggesting that at this temperature, its turnover is faster than at 10°C.

To understand the molecular mechanisms that are involved in *rhlE* induction at low temperature, transcriptional fusions to the *lacZ* reporter gene were used. The 5′ untranslated region (UTR) of the *rhlE* mRNA is 186 nucleotide (nt) long (31) and is predicted to have several stem-loops that could have a role in regulating mRNA stability (Fig. 4B). In order to test whether the presence of the 5′ UTR was important for expression, we used two constructs (Fig. 4C, fusions 1 and 2), one containing both the promoter and the 5′ UTR and another containing the promoter region only. Gene expression was determined by measuring the β-galactosidase activity of the constructs in the wild-type NA1000 strain and in the *rhlE* mutant both at 30°C and 10°C (Fig. 4D). Although we could not observe an induction of transcription at 10°C in the wild-type strain, expression was higher in the *rhlE* null strain at both temperatures, indicating that RhlE could be regulating its own mRNA levels. Moreover, the constructs lacking the 5′ UTR showed a reduction in expression in both strains (approximately 30% at 30°C and 55% at 10°C in NA1000, and 50% at 30°C and 70% at 10°C in the *rhlE* mutant). These results indicate that the 5′ UTR is necessary for maximal expression of the *rhlE* gene, probably due to mRNA stabilization, since the translation of the *lacZ* gene in these constructs was determined by the vector sequences (Fig. 4C, fusions 1 and 2). To evaluate whether *rhlE* could be regulated at the translational level, an integrated translational fusion of this element to the *lacZ* gene was used (Fig. 4C, fusion 3). The results showed that while expression levels were constant at 30°C, there is a large increase in β-galactosidase activity after 2 h at 10°C (Fig. 4E), indicating that the increase in protein levels observed could be at least in part attributed to posttranscriptional mechanisms.

### RhlE is associated with the RNA degradosome.

**FIG 5:**
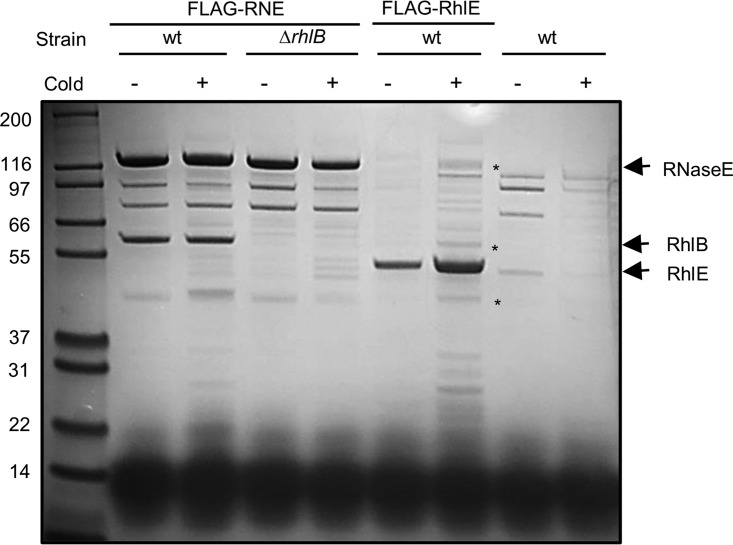
Participation of RhlE in the C. crescentus RNA degradosome. Cultures of C. crescentus were grown at either 30°C (cold−) or 15°C (cold+) up to mid-log phase, and RNA degradosomes were isolated. The strains used were NA1000 (wt) and MM71 (Δ*rhlB*) containing FLAG-tagged RNase E (FLAG-RNE), MM84 (NA1000 containing FLAG-tagged RhlE), and NA1000 with no tagged proteins as a negative control. Asterisks indicate components of C. crescentus degradosome identified by mass spectrometry that copurified with FLAG-RhlE: RNase E, RhlB, and methionine adenosyltransferase, respectively, from top down.

The RNA helicase RhlB was previously identified as a component of the C. crescentus degradosome at 30°C (28). In E. coli, the DEAD-box RNA helicase CsdA was also shown to associate with the degradosome at low temperature (25), and we explored if alternative helicases might associate with the C. crescentus degradosome at low growth temperature. A strain carrying the RNase E protein with an amino-terminal FLAG epitope was used in pulldown assays to identify new proteins that copurify with the degradosome at 15°C. As shown in Fig. 5, more proteins copurified with RNase E at 15°C than at 30°C, indicating that other components may associate with the degradosome under this condition. The same assay was carried out with the *rhlB* deletion mutant and, except for the RhlB band, no major difference was observed compared to the wild-type strain, suggesting that the loss of RhlB is not compensated by replacement with an alternative helicase at low temperature. To verify whether the RhlE protein could interact directly with the RNA degradosome components, a strain carrying a FLAG-RhlE protein was constructed. Pulldown assays at both temperatures showed that in addition to an increase in the amount of RhlE recovered at 15°C, many additional proteins were copurified with FLAG-RhlE, including some bands showing the same migration as the RNA degradosome components. Bands were excised from the gel and identified by mass spectrometry as RNase E and RhlB, showing that RhlE can in fact associate with the degradosome at 15°C. A third excised band corresponds to methionine adenosyltransferase, which also appears to associate with the degradosome (see below) (Fig. 5).

**FIG 6:**
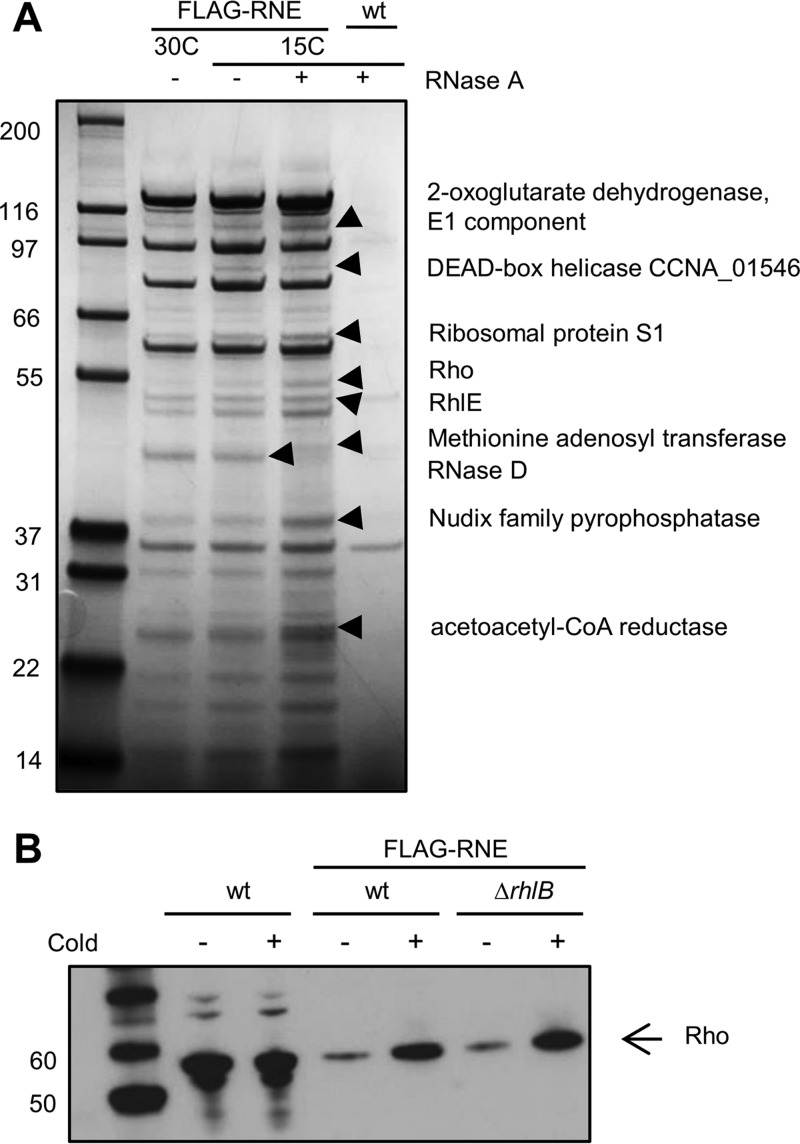
Characterization of proteins associated with the C. crescentus RNA degradosome at low temperature. (A) Cultures of C. crescentus were grown at either 30°C or 15°C up to mid-log phase, and RNA degradosomes were isolated. The strains used were NA1000 containing FLAG-tagged RNase E (FLAG-RNE) and NA1000 with no tagged proteins (wt) as a negative control. The proteins indicated by arrowheads were identified by mass spectrometry. (B) Association of Rho with the C. crescentus RNA degradosome. Cultures of C. crescentus were grown at either 30°C (cold−) or 15°C (cold+) up to mid-log phase, and proteins from total cell extracts or isolated RNA degradosomes were separated by SDS-PAGE. The proteins were transferred to a PVDF membrane, and the Rho protein was identified by immunoblotting with an anti-Rho serum. The samples used were total extract of NA1000 (wt) and isolated RNA degradosomes from NA1000 (wt) or MM71 (Δ*rhlB*) containing FLAG-tagged RNase E (FLAG-RNE).

Further comparison of the composition of the RNA degradosome at 15°C and 30°C was performed using the FLAG-RNase E strain at both temperatures and also by pretreating the cell extracts at 15°C with RNase A to eliminate interactions mediated by RNA. Figure 6 shows that all the main degradosome bands remain after the RNase treatment, except for the RNase D band that is less intense in the RNase A-treated sample. This suggests that all the minor proteins that copurify with the RNA degradosome are doing so by protein-protein interaction. Several of the copurifying bands were excised from the gel and identified by mass spectrometry, and the results showed several enzymes that are potentially associating with the RNA degradosome *in vivo*. Some metabolic enzymes were found: the tricarboxylic acid (TCA) cycle enzyme 2-oxoglutarate dehydrogenase E1 component (CCNA_00342), the dicarboxylate metabolism enzyme acetoacetyl coenzyme A (acetoacetyl-CoA) reductase (CCNA_00545), a NUDIX family pyrophosphatase (CCNA_00267), which converts NAD^+^ into nicotinamide d-ribonucleotide, and a methionine adenosyltransferase (CCNA_00048). Proteins involved in RNA metabolism were also present, such as the small subunit ribosomal protein S1 (CCNA_03702), the transcription terminator Rho (CCNA_03876), and two DEAD-box RNA helicases, RhlE and CCNA_01546.

The results showed that RhlE is associating with the RNA degradosome, not only at 15°C but also at 30°C (Fig. 6A). However, the RhlB protein is not displaced by the presence of RhlE, suggesting that RhlE could bind to the RNase E scaffold in a different site or that its interaction is indirect, being mediated by another protein. Nevertheless, RhlE is not dependent on RhlB to associate, since it is still pulled down in the *rhlB* mutant (Fig. 5).

The identification of the Rho transcription termination factor in these experiments may have mechanistic implications. C. crescentus Rho is autoregulated by antitermination of transcription and is regulated during the cell cycle (32), but its expression was not found to be cold induced (data not shown); however, its presence in the RNA degradosome seems to increase at low temperature (Fig. 6A). To establish if this is the case, the pulldown experiments were repeated, and the Rho presence was more clearly confirmed by immunoblot analysis with an anti-Rho serum (Fig. 6B). We can observe that there are equal amounts of Rho protein in the cell extracts at both 30°C and at 15°C, confirming that the protein is not cold induced. However, more Rho protein seems to be associated with the RNA degradosome at 15°C, indicating that there is increased recruitment to the complex at low temperature. The absence of RhlB does not change the result (Fig. 5), indicating that the proteins do not compete for the same binding site.

## DISCUSSION

In this work, we have characterized the DEAD-box RNA helicase RhlE and showed that it is an important enzyme for cell fitness and is induced upon temperature decrease from 30°C to 15°C. The absence of RhlE leads to a growth defect that is accentuated at low temperature. While the *rhlB* deletion alone does not compromise growth at either temperature, the *rhlE rhlB* double mutant shows a more severe growth phenotype at 15°C, indicating that both RNA helicases are necessary for growth and adaptation to low temperature. The double mutant displays an aberrant morphology at 15°C, with elongated cells that suggest a defect in cell division, which is consistent with its growth phenotype. Interestingly, the overexpression of RhlB does not complement the *rhlE* knockout, suggesting that RhlE has a cellular role that is not fulfilled by RhlB.

The transcription initiation site for *rhlE* was predicted to be 186 bp upstream of the start codon (31). We have determined that *rhlE* maximal expression is dependent on its long 5′ UTR and that RhlE is regulated by posttranscriptional mechanisms. The regulation of other cold-induced genes was shown to be dependent on their long 5′ UTR, such as the C. crescentus cold shock proteins CspA and CspB (33). Cold induction of C. crescentus *cspA* was shown to be a result of transcription attenuation at optimal temperature, and the translation of both genes was enhanced at low temperature (33). The 5′ UTRs of C. crescentus *cspA* and *cspB* mRNAs share a region of sequence similarity (called the upstream box) necessary for cold induction, but this sequence is not present in the 5′ UTR of *rhlE* (data not shown). The results indicated that RhlE could be regulating its own mRNA levels, either by mRNA stabilization mediated by the 5′ UTR and/or by enhancement of translation. Several bacterial DEAD-box RNA helicases have been shown to be induced by cold shock (34–38). The mechanisms of regulation of the cyanobacterial genes for RNA helicases have been studied in more detail; the reports showed that *crhC* from Anabaena sp. is regulated at the levels of transcription, mRNA stability, and translation (35) and that the CrhR RNA helicase from Synechocystis is regulated by proteolysis (39). The utilization of several levels of control in the expression of RNA helicases seems to be a recurrent feature in bacteria from distant phylogenetic lineages, indicating the important role of these enzymes at low temperature.

We have shown that two further DEAD-box RNA helicases are able to associate with RNase E in addition to the canonical RhlB protein, namely, RhlE and the gene product of CCNA_1546. RhlB was shown to bind to the S1 and sensor domain at the amino-terminal domain of C. crescentus RNase E (40). Our results showed that RhlB is not displaced from the degradosome by the presence of RhlE, and the absence of RhlB does not increase the amounts of RhlE associated with the assembly (Fig. 5). These results suggest that RhlE could be binding to RNase E at a different site from RhlB. However, *in vitro* RNase E and RhlB form a complex of one tetramer to two RhlB monomers (40), and *in vivo* ribosome profile data suggest the proteins are present in the cell at the same 1:2 tetramer-to-monomer ratio (41). This stoichiometry supports the possibility that RhlE could bind to the two sites unbound by RhlB on every degradosome tetramer, so additional binding of RhlE might not displace RhlB in this event. The incorporation of multiple helicases into the C. crescentus RNA degradosome is perhaps not surprising, as other RNA helicases were shown to associate with the degradosome in E. coli (18, 25), Pseudomonas syringae (42), and the alphaproteobacterium Rhodobacter capsulatus (43).

Other types of RNA helicases were also found to become part of the C. crescentus low-temperature RNA degradosome. An interesting result was the identification of the DNA:RNA helicase Rho associating with the C. crescentus RNA degradosome at low temperature. In C. crescentus, the RNA degradosome appears to localize in the nucleoid region, suggesting that active transcription could be coupled with RNA degradation (44). Rho was also found to be part of the R. capsulatus RNA degradosome (43), and its presence in the degradosome was shown to fluctuate with environmental conditions, increasing when cells were under high oxygen tension (45). We can speculate that in alphaproteobacteria, Rho may have a more prominent role in RNA processing under stress conditions, perhaps facilitating the termination of transcription with degradation of mRNA by the degradosome. Moreover, such a process could be mediated by regulatory RNAs (46), which might be corecruited by RNase E and chaperones.

The RNA degradosome coprecipitations identified the presence of the small subunit ribosomal protein S1, which also has an unwinding RNA activity (47, 48). S1 was found to interact directly with RNase E and PNPase in E. coli (49), and it was previously suggested that the S1 protein may stabilize mRNA and initiate translation, protecting transcripts from degradation under conditions of impaired translation, such as in cold shock (50). Moreover, a loss-of-function mutation in ribosomal protein S1, yielding a protein lacking one of its four RNA binding domains was identified as a suppressor for a RNase E temperature-sensitive (TS) mutation that affects its mRNA turnover ability (51). In fact, the E. coli RNA degradosome was found to directly interact with ribosomes, indicating that translation and mRNA degradation may be coupled, possibly mediated by small RNA (sRNA) and Hfq (52).

In conclusion, in this work, we have shown that the DEAD-box RNA helicase RhlE is essential for cell fitness in C. crescentus at low temperature and is induced under this condition, mainly through posttranscriptional regulation. RhlE associates with the RNA degradosome at low temperature, together with several other proteins, such as a second DEAD-box RNA helicase, the transcriptional terminator Rho, and ribosomal protein S1, all of which can have accessory roles in RNA degradation at low temperature.

## MATERIALS AND METHODS

### Construction of C. crescentus strains and growth conditions.

C. crescentus NA1000 (53) was used as the original strain for the construction of all mutant strains (Table 1). E. coli DH5α was used for all cloning purposes (Table 1). The *rhlB* deletion strain was generated by amplification of the gene-flanking regions by PCR with primer pairs CC1847-C/CC1847-D (upstream fragment) and CC1847-E∕CC1847-F (downstream fragment) (Table 1). The fragments were digested with ApaI and BamHI (upstream fragment) and BamHI and EcoRI (downstream fragment) and cloned in tandem in pNPTS138 suicide vector (M. R. K. Alley, unpublished data) digested ApaI and EcoRI. To generate the *rhlB rhlE* double mutant, the *rhlE*::mini-Tn*5* mutation from clone 30-10H (29) was transduced with phage ϕCr30 (54) into the MM50 strain. In order to warrant a similar genetic background, the mutation was also moved by transduction into a fresh NA1000 strain. The deletion of *rhlB* in a strain with the FLAG-RNase E protein was obtained by introducing the pNPTS138 vector for *rhlB* deletion into the NA1000 (FLAG-RNase E) strain (55). The resulting strains were named MM50 (NA1000 Δ*rhlB*), MM74 (NA1000 *rhlE*::mini-Tn*5*), MM71 (NA1000 Δ*rhlB* FLAG-RNE), and MM82 (NA1000 Δ*rhlB rhlE*::mini-Tn*5*).

**TABLE 1:** Strains, plasmids, and oligonucleotides used in this study

Strain, plasmid, or primer	Description or sequence^*a*^	Reference, source, or purpose
Strains		
Caulobacter crescentus		
NA1000	Synchronizable derivative of CB15	53
NA1000 FLAG-RNase E	NA1000 expressing FLAG-RNase E	55
MM50	NA1000 Δ*rhlB*	This work
MM71	NA1000 FLAG-RNase E Δ*rhlB*	This work
MM74	NA1000 *rhlE*::mini-Tn*5*	29
MM82	NA1000 Δ*rhlB rhlE*::mini-Tn*5*	This work
MM84	NA1000 FLAG-RhlE	This work
Escherichia coli		
DH5α	(ϕ80*lacZ*ΔM15) *hsdR17 recA1 endA1 gyrA96 thi*-*1 relA1*	63
S17-1	294::RP4-2(Tc::Mu)(Km::Tn*7*)	56
Plasmids		
pGEM	Cloning vector, Ap^r^	Promega
pNPTS138	Suicide vector containing *oriT* and *sacB*, Kan^r^	M. R. K. Alley, unpublished data
pET-15	Cloning vector	Novagen
pET-DUET	Cloning vector	Novagen
pBV-MCS4	Cloning vector	58
pRKlacZ290	Reporter vector for transcriptional fusions to *lacZ*, Tet^r^	59
pJBZ281	Reporter vector for translational fusions to *lacZ*, Kan^r^	60
Primers		
CC1847-C	TTGGGCCCCGTCGAAGGCGGGAGTCGTG	Deletion of *rhlB*
CC1847-D	TTTAAGCTTGGAGCCGCCCGTGCTTGG	Deletion of *rhlB*
CC1847-E	TTTAAGCTTTCGGCAAGATCCATCGAGG	Deletion of *rhlB*
CC1847-F	TTGAATTCCGGTGCGGTGTTCATGCCCT	Deletion of *rhlB*
RhlE1	TGAATTCGACTGAAGATGACGGTTTCG	Amplification of *rhlE* promoter for transcriptional fusion
RhlE2	TGGATCCGGTCGGTTCCTTGTAGC	Amplification of *rhlE* promoter for transcriptional fusion
P0835-1	TGGATCCGGCGCGGGGTCCGTATCGG	Amplification of *rhlE* promoter without 5′ UTR for transcriptional fusion
RhlE 10	CCATGGACTACAAGGACGACGACGACAAGGTGACTCAATTTTCCGACCTTGG	Construction of FLAG-RhlE strain
RhlE 11	CTTGTCGTCGTCGTCCTTGTAGTCCATGGGTGTTGTGTCTTTCGTGCGCC	Construction of FLAG-RhlE strain
RhlE 12	AAAAGAATTCGTGGCAGCTTGGCCATCTCCG	Construction of FLAG-RhlE strain
RhlE 13	AGCTGCTTGTCGTTGCGACGG	Construction of FLAG-RhlE strain
RhlE-tra	AGGATCCACGTGTGTTGTGTCTTTCG	Amplification for *rhlE* translational fusion
ccRhlB-DUET.f	GCCATATGACTGAATTCACCGACC	Amplification of *rhlB* for cloning
ccHel.REV2	GCCTCGAGCTACTTCGCGCCGCGCGGCGGCCGCGCGAGG	Amplification of *rhlB* for cloning
cc0835_Nde.F	CAGCCATATGACTCAATTTTCCGACCTTGGCCTG	Amplification of *rhlE* for cloning
cc0835_Bam.R	GCCGGATCCTTAGTCGATAGGCGACCAGCG	Amplification of *rhlE* for cloning

a Underlined letters indicate restriction enzyme recognition sites used for cloning purposes. Ap^r^, ampicillin resistance; Kan^r^, kanamycin resistance; Tet^r^, tetracycline resistance.

The FLAG-RhlE strain was generated by amplification of the *rhlE* gene with primers that introduced a single FLAG peptide at the amino terminus (primer pairs RhlE12/RhlE11 and RhlE10/RhlE13). The two fragments were sequentially cloned into pNPTS138 using the restriction enzymes EcoRI and NcoI, and NcoI and ApaI, respectively. The vectors were introduced into E. coli S17-1 (56) and transferred into C. crescentus NA1000 by conjugation. After integration, the strains were grown in PYE medium (57) containing 3% sucrose, and the second recombination event was screened by PCR. The resulting strain was named MM84 (NA1000 [FLAG-*rhlE*]).

The strains were grown in PYE medium and incubated at 30°C or 15°C with agitation. When necessary, antibiotics were added at the following concentrations: tetracycline, 1 μg/ml; kanamycin, 5 μg/ml; nalidixic acid, 20 μg/ml; or gentamicin, 5 μg/ml. Escherichia coli strains were grown at 37°C in Luria-Bertani (LB) medium. When necessary, antibiotics were added at the following concentrations: tetracycline, 12.5 μg/ml; ampicillin, 100 μg/ml; or kanamycin, 50 μg/ml. Generation times were calculated from four individual growth curves for each strain.

### Microscopy.

Overnight cultures were diluted in PYE medium to an optical density at 600 nm (OD_600_) of 0.1 and incubated at 30°C or 15°C with agitation until an OD_600_ of 0.5 was reached. Samples were collected and mounted on slides with agarose pads (1% agarose in phosphate-buffered saline). Microscopy was performed with a Nikon E800 microscope equipped with 100× differential interference contrast (DIC) (Nikon Instruments, Inc., Melville, NY). Images were captured with a Nikon DS-Ri1 camera and were analyzed with the UVP Doc-ItLS software (Life Science Software).

### Construction of complementing plasmids.

To obtain the complementation of *rhlB* and *rhlE* deletions, the coding regions of each gene were initially amplified with primer pairs cc0835_Nde.F/cc0835_Bam.R (*rhlE*) and ccRhlB-DUET.f/ccHel.REV2 (*rhlB*) and cloned into pET15 and pET DUET, respectively. Each fragment was isolated by digestion with NdeI and BamHI (*rhlE*) or NdeI and XhoI (*rhlB*) and was cloned into the pBV-MCS-4 vector (58) digested with the respective enzymes. The plasmids were introduced into C. crescentus strains by conjugation with E. coli S17-1 and selected in PYE-gentamicin-nalidixic acid plates.

### *rhlE* expression assays.

Transcriptional fusions to the *lacZ* reporter gene were constructed in plasmid pRKlacZ290 (59), and translational fusions were made in plasmid pJBZ281 (60). The transcriptional fusions were made by amplifying the promoter region with the 5′ UTR with primer pair RhlE1/RhlE2 and the promoter region alone with primer pair RhlE1/P0835-5 (Table 1). For the translational fusion, a 450-bp fragment corresponding to the *rhlE* promoter and 5′ UTR regions was amplified with primers RhlE1 and RhlE-tra, cloned in pGEM-T Easy, and confirmed by DNA sequencing. The fragment was isolated by digestion with EcoRI and BamHI and cloned in frame with *lacZ* into pJBZ281 digested with the same enzymes. The pRKlacZ290 derivatives were introduced into C. crescentus NA1000 and MM74 by conjugation with E. coli S17-1. The pJBZ281 derivative was introduced into NA1000 by electroporation, and chromosomal integration was selected in PYE-kanamycin plates. Gene expression was measured by determining the β-galactosidase activity, as described by Miller (61). β-Galactosidase activity assays were performed with cultures at the mid-log phase from C. crescentus NA1000 strain at 30°C and at different time points during incubation at 10°C.

### Purification of FLAG-tagged proteins.

C. crescentus strains (FLAG-RNE/FLAG-RhlE) were grown asynchronously in 1 liter of PYE medium per pulldown experiment to an OD_600_ of 0.4 at 30°C. For cold treatments, cells were grown in 1 liter of PYE to an OD_600_ of 0.2 at 30°C before the temperature was lowered to 15°C and growth continued for a further 5 h, resulting in a final OD_600_ of ∼0.4. Cells were then harvested by centrifugation, resuspended in 30 ml of lysis buffer (20 mM Tris-HCl [pH 7.5], 250 mM NaCl, 5 mM EDTA), flash-frozen, and stored at −80°C until required. Cell pellets were thawed on ice and then lysed by two passages through a high-pressure homogenizer at 50,000 kPa (EmulsiFlex; Avestin). The lysate was clarified by centrifugation (30 min, 30,000 × *g*, 4°C), and the supernatant was incubated with 50 μl of anti-FLAG M2 affinity gel per pulldown experiment (Sigma-Aldrich) prewashed in lysis buffer (2 h, 4°C). The anti-FLAG affinity gel was collected by gravity sedimentation, transferred to a minichromatography column (Thermo Scientific), and washed three times with 500 μl of lysis buffer. To elute bound proteins, the anti-FLAG resin was incubated with 30 μl of FLAG peptide diluted to 0.25 mg/ml in elution buffer (20 mM Tris-HCl [pH 7.5], 100 mM NaCl) for 10 min at 4°C, and the eluate was collected by centrifugation and analyzed by SDS-PAGE using a 4 to 12% polyacrylamide NuPAGE gel (Thermo Scientific). Protein bands were identified by in-gel matrix-assisted laser desorption ionization (MALDI) mass spectrometry fingerprinting (PNAC facility, Department of Biochemistry, University of Cambridge).

### Immunoblots.

Proteins were separated by SDS-PAGE in either 10% polyacrylamide gels or 4 to 12% polyacrylamide NuPAGE gels (Invitrogen), electrotransferred to cellulose or polyvinylidene difluoride (PVDF) membranes, and treated as described by Towbin et al. (62). For the detection of Rho, the membranes were blocked overnight with Tris-buffered saline (TBS) with 1% milk at 4°C and then incubated with anti-Rho antiserum (32) in a 1:1,000 dilution in TBS-milk for 2 h at room temperature (RT). After washing, the membrane was incubated with horseradish peroxidase-conjugated secondary anti-rabbit IgG at a 1:5,000 dilution (Sigma), and the bands were developed with the EZ-ECL reagent (Biological Industries), as indicated by the manufacturer.

For the detection of FLAG-RhlE, the membranes were incubated for 16 h with anti-FLAG monoclonal antibody (Sigma) at a 1:20,000 dilution in TBSTT (10 mM Tris-HCl [pH 8.0], 150 mM NaCl, 0.03% Tween 20, 0.02% Triton X-100), followed by incubation with alkaline phosphatase-conjugated anti-mouse IgG (Sigma) at a 1:5,000 dilution in TBS containing 5% milk. As a control, anti-Fur polyclonal antiserum 1:1,000 dilutions were used, followed by alkaline phosphatase-conjugated anti-rabbit IgG (Sigma) diluted 1:5,000 in TBS. Bands were developed with 0.5 mg/ml nitroblue tetrazolium (NBT) and 0.15 mg/ml 5-bromo-4-chloro-3-indolylphosphate (BCIP) in alkaline phosphatase buffer (100 mM Tris-Cl [pH 9.5], 5 mM MgCl_2_, 100 mM NaCl).

## Supplementary Material

Supplemental material
