# Supplementary material for: Association of the Cold Shock DEAD-Box RNA Helicase RhlE to the RNA Degradosome in Caulobacter crescentus
Source: J Bacteriol. 2017 Jun 13;199(13):e00135-17. doi: 10.1128/JB.00135-17 (PMC5472812; doi:10.1128/JB.00135-17)
Supplement: Supplemental material [file supp_199_13_e00135-17__index.html]

Supplemental material 

# Association of the Cold Shock DEAD-Box RNA Helicase RhlE to the RNA Degradosome in Caulobacter crescentus

## Supplemental material

- Supplemental file 1 -

  Fig. S1 (Cell viability of *C. crescentus* RNA helicase mutant strains at low temperature), S2 (Expression profile of RhlE), S3 (Accumulation of RhlE at 10ºC), and S4 (Nucleotide sequences of the 5′-UTRs of *cspA*, *cspB*, and *rhlE*)

  PDF, 323K
