# Supplementary material for: Association of the Cold Shock DEAD-Box RNA Helicase RhlE to the RNA Degradosome in Caulobacter crescentus
Source: J Bacteriol. 2017 Jun 13;199(13):e00135-17. doi: 10.1128/JB.00135-17 (PMC5472812; doi:10.1128/JB.00135-17)
Supplement: Supplemental material [file JB.00135-17_zjb999094430s1.pdf]

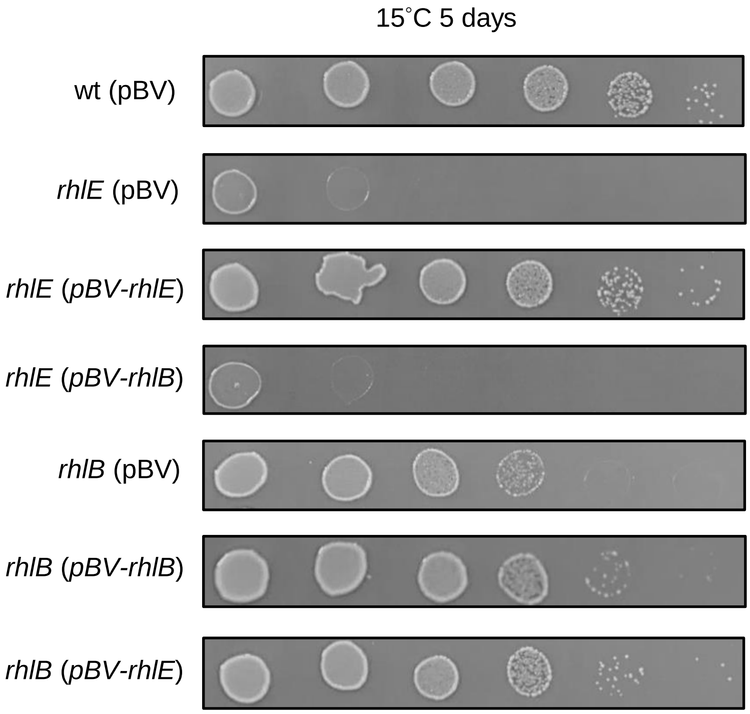

Fig. S1. Cell viability of C. crescentus RNA helicases mutant strains at low temperature. (A) Serial dilutions of the cultures at OD 0.1 (10^{-1} to 10^{-5} in 10 μl) were plated in PYE medium and plates were incubated at 15C for 5 days. Strains genotypes are indicated in each panel, for the respective strain: NA1000 (wt), MM24 (*rhlE*::Tn5), MM50 (Δ*rhlB*), MM82 (*rhlE*::*Tn5*-Δ*rhlB*). The strains carried the pBV empty vector (pBV) or the vector containing a copy of the indicated gene.

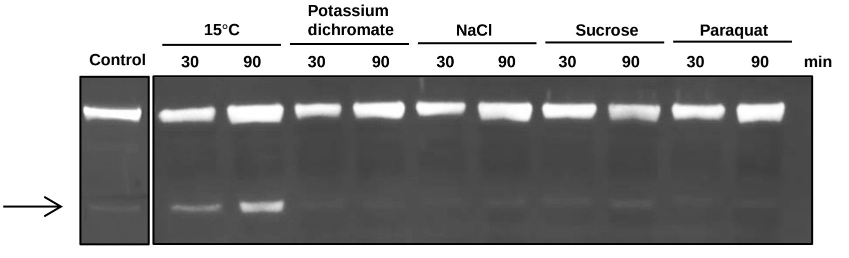

Fig. S2. Expression profile of RhlE. A culture of *C. crescentus* strain MM84, containing a tagged FLAG-RhlE protein, was incubated at 30ºC up to midlog phase (control) and then divided in aliquots that were incubated with each stressing agent for 30 and 90 minutes. Samples were taken at the indicated times and protein accumulation was evaluated by immunoblotting using an anti-FLAG antibody. The arrow indicates the RhlE protein. Stressing agents were: potassium dichromate (55 µM), NaCl (85 mM), sucrose (150 mM), and paraquat (50 µM). A non-specific protein identified by a second antiserum is shown above as a control for protein loaded in each lane.

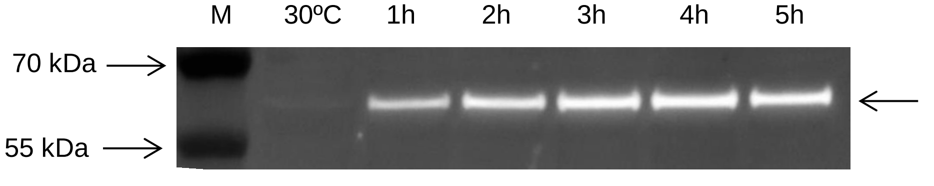

Fig. S3. Accumulation of RhlE at 10ºC. A culture of *C. crescentus* strain MM84, containing a tagged FLAG-RhlE protein, was incubated at 30ºC up to midlog phase (30ºC) and then transferred to 10ºC. Samples were taken at the indicated times and protein accumulation was evaluated by immunoblotting using an anti-FLAG antibody. The arrow indicates the FLAG-RhlE protein. M, molecular weight marker.

rhlE – 5′– CAUUCCGGCGCUAUUGGCGCUCCAGCCGCGUGAGGGUCCGUUUUCGGACCAGCCUGUCGAGC

cspA – 5′– GGCCUGCUUUUCCUUGCUCCUGGACCAGGAGCGCCGGACGCGCCUACCGAACUUCCA
cspB – 5′– CUCGGUCGCUCUCCACGCUUGCUCUUCGGAUCGAGCUUCGGGCCUACCAAGAACCU
rhlE – GCCUUGAGAGCUUUUCUCACCCGAUCGCCCGUGCACGCGGGGCGCUCCCCCGAACGA

Upstream Box RBS

cspA – ACGAAAUUUGAUCGUCGACCGGGCGUUCCGGACGACGAACACCUAUGCCUACAAGGAACAUCCCC**AUG**
cspB – CUCCGACAAUUUGAUCGUAGGGCCUGUUUGAGGCCUCGCGUCACCACAUCUGAAAGAUACAAAC**AUG**
rhlE – UAGCCGACAGGCCAGCCGUGCGAGAAGCGCAUGGGCUGCCUUUCGGCGCACGAAAGACACAACACAC**GUG**

Fig S4. Nucleotide sequences of the 5'-UTRs of *cspA, cspB* and *rhlE*. The 13 nt sequence shared by *cspA* and *cspB* called Upstream Box is shaded. The ribosome binding site is boxed.
